# The Predictive Value of Three Variables in Patients with Metastatic Renal Cell Carcinoma Treated with Immune-Based Combination Therapies in Randomized Clinical Trials: A Systematic Review and Meta-Analysis

**DOI:** 10.1155/2022/7733251

**Published:** 2022-09-10

**Authors:** Min Hou, Haiyan Xing, Shuangshuang He, Xue Yang, Dan Peng, Yang Li, Qing Zhang, Pan Zhang, Yunqi Ma, Juan Li, Jinlu Shan, Yao Liu

**Affiliations:** ^1^Department of Pharmacy, Daping Hospital, Army Medical University, Chongqing 400042, China; ^2^Cancer Center, Daping Hospital, Army Medical University, Chongqing 400042, China

## Abstract

**Background:**

Sex, age, and International Metastatic Renal-Cell Carcinoma Database Consortium (IMDC) prognostic risk may influence the immune response. Nonetheless, the correlation between these factors and the survival benefits of immune-based combination therapies in patients with metastatic renal cell carcinoma (mRCC) is controversial and undefined. As a result, the purpose of this research is to evaluate the potential differences of immune-based combination therapies on survival benefits from mRCC subgroups.

**Methods:**

PubMed, Cochrane Library, Embase, and http://www.clinicaltrials.gov were searched from inception to March 17, 2022. Randomized clinical trials (RCTs) comparing overall survival (OS) or progression-free survival (PFS) in patients with mRCC treated by immune-based combinations vs. contemporary first-line therapies were included.

**Results:**

Five RCTs with a total of 4206 subjects were included. An OS and PFS benefit of immune-based combinations were found for patients of different sex, age, and IMDC intermediate/poor risk. No obvious difference in relative PFS benefit from immune-based combinations over the control group was found in patients of different genders (*P*=0.71, I^2^ = 0%), ages (*P*=0.55, I^2^ = 0%), or IMDC prognostic risks (*P*=0.38, I^2^ = 0%). However, the difference in OS benefit was significant regarding age (*P*=0.009, I^2^ = 85.5%) and IMDC prognostic risk (*P*=0.004, I^2^ = 82.2%).

**Conclusions:**

This meta-analysis found that immune-based combination therapies should not be restricted to certain patients with mRCC in gender categories. However, age and IMDC prognostic risk of mRCC patients are associated with different outcomes of OS and thus help identify those patients most probably to benefit from immune-based combination therapies.

## 1. Introduction

Renal cell carcinoma (RCC) is by far the most common type of kidney cancer, with increased incidence and death rates in 195 countries from 1990 to 2017, which has been found to place a large economic burden on society [1]. There were 431,228 cases of kidney cancer diagnosed worldwide and about 179,368 deaths from the disease in 2020, corresponding to 2.2% and 1.8% of all cancers, respectively [2]. Up to 17% of patients present initially with metastatic disease [3], with a 12% five-year survival rate [4].

Metastatic RCC (mRCC) is one of the most difficult malignancies to treat, and the effects of chemotherapy and surgery are limited or ineffective [5]. New targeted therapies (including kinase inhibitors, the kinase mammalian target of rapamycin (mTOR) inhibitors, and monoclonal antibodies against vascular endothelial growth factor (VEGF)) have been recommended as first-line treatment, and immune-based combination therapies have been more and more applied in mRCC treatment in the past 5 years. Recent clinical trials have demonstrated that immune-based combination therapies have survival benefits in comparison with sunitinib in the first-line therapy of mRCC [6–12]. Despite these promising advances, selecting which treatment approaches to use for any given patient remains a significant challenge.

Patients with different immune responses may get various benefits from immune-based combination therapies. As is known to all, women have stronger adaptive and innate immune responses than men [13]. Sex differences in cancer immunotherapy are just starting to be revealed. Conforti et al. confirmed that, in contrast to women, men benefit more from cancer immunotherapy [14]. Globally, RCC is the sixth most prevalent cancer in men and the ninth in women, which respectively account for 5% and 3% of all tumor diagnoses [15], and men are diagnosed with RCC at almost twice the rate of women. The above findings may suggest greater benefits to male patients with mRCC. However, conflicting results were reported in recent studies, which showed that there was no statistically significant correlation between the gender of patients and the benefit of immunotherapy for advanced cancer [16, 17]. There existed a certain association between the effect of tumor immunotherapy and the age of patients; it has been hypothesized that older patients benefited less from immunotherapy than younger patients due to declining immune cells within the tumor microenvironment, but current research conclusions were controversial [18, 19]. Furthermore, these results are derived from the comparison between the intervention group with combined chemotherapy or single immune-checkpoint inhibitor (ICI) treatment and the control group not treated by ICIs. However, little is known about the impact of patient gender on the efficacy of ICI combination with ICI or targeted therapy (TT) as cancer treatments. The IMDC prognostic models are the current standard for mRCC patients' stratification, which provides information for patient consultation and treatment choice to a certain extent [20]. According to the IMDC prognostic model, mRCC patients can be classified as poor, moderate, or favorable risk, which has different biological characteristics. Some evidence shows that stratified treatment is needed. The favorable-risk patients are more suitable for targeted therapy, while the intermediate and poor-risk patients may need combined immunotherapy.

Data from phase III studies showed different survival benefits regarding sex, age, and IMDC prognostic risk association with immune-based combination therapies for patients with mRCC. However, up to now, there has been no systemic evaluation of the different magnitudes of survival benefits from it. Therefore, in this paper, we performed a systematic review and meta-analysis to evaluate the potential association of sex, age, and IMDC prognostic risk with immune-based combination therapies' survival benefits in patients with mRCC.

## 2. Methods

In accordance with the Preferred Reporting Items for Systematic Reviews and Meta-analyses (PRISMA) extension statement [21], the systematic review and meta-analysis of randomized clinical trials (RCTs) for the mRCC with immune-based combinations treatment were conducted.

### 2.1. Search Strategy

Two reviewers (including MH and HYX) searched PubMed, Cochrane Library, Embase, and http://www.clinicaltrials.gov independently for articles by entering “renal cell cancer” or “renal cell carcinoma” or “kidney carcinoma” or “kidney cancer” and “advanced” or “metastatic” and “randomized” as the search terms. Subsequently, check all the articles as well as their reference lists with the aim of expanding the underlying related articles. Search the database from inception to March 17, 2022. The primary outcomes, including overall survival (OS) and progression-free survival (PFS), stratified by patient sex, age, and IMDC prognostic risk were collected.

### 2.2. Inclusion and Exclusion Criteria

If the articles accord with these criteria, they were included in this meta-analysis: (1) RCTs assessing survival benefits of immune-based combinations for mRCC patients; (2) the control and treatment groups were given contemporary first-line therapies and ICI–ICI/TT therapies, respectively; and (3) the article provides associated indicators of survival benefits (PFS or OS) or can be counted based on the original data. When one of these issues occurs, the article should be excluded: (1) the subjects were animals; (2) the controls were placebo or interferon; and (3) other kinds of trials such as observational studies, crossover designs, healthy controlled trials, self-contrast trials, letters, editorials, reviews, meeting abstracts, and case reports.

### 2.3. Data Extraction

Two investigators (SSH and XY) independently extracted the following information from the included articles: first author's name, publication year, national clinical trial number, period of patient recruitment, study design, number of patients, age, sex, and survival outcomes. Subsequently, hazard ratios (HRs) and 95% confidence intervals (CIs) related to OS and PFS were retrieved. Disagreement between the two researchers was resolved through negotiation or consensus with the third author (MH or HYX).

### 2.4. Risk of Bias Assessment

In accordance with the Review Manager Software version 5.3 (RevMan 5.3) afforded via Cochrane Collaboration, two independent reviewers (SSH and XY) utilized a seven-item scale [22] to evaluate the risk of bias existing in the included research. Each of the items involves assigning a judgment of high, low, or unclear risk of material bias, and a lower bias exhibits better quality. The Cochrane Handbook provides specific criteria for judging the risk of bias from each item. Any differences were resolved through consultation with the coauthor (YL).

### 2.5. Statistical Analyses

RevMan 5.3 was applied for synthesizing all the extracted data, OS and PFS benefit of immune-based combinations for each subgroup of mRCC patients were calculated by HRs with 95% CIs with a fixed- or random-effect model, i.e, men vs. women, younger (<65 years) vs. older (≥65 years), and patients with IMDC prognostic risk (favorable vs. intermediate vs. poor). A chi-square test was utilized for heterogeneity analysis. If I^2^ is below 50%, there is no evident heterogeneity, which is acceptable. As a result, the fixed-effect model of analysis is suitable. Otherwise, the random-effect model is considered [23, 24]. The publication bias was evaluated through the funnel plots.

## 3. Results

### 3.1. Search Results and Patient Characteristics

**Figure 1:**
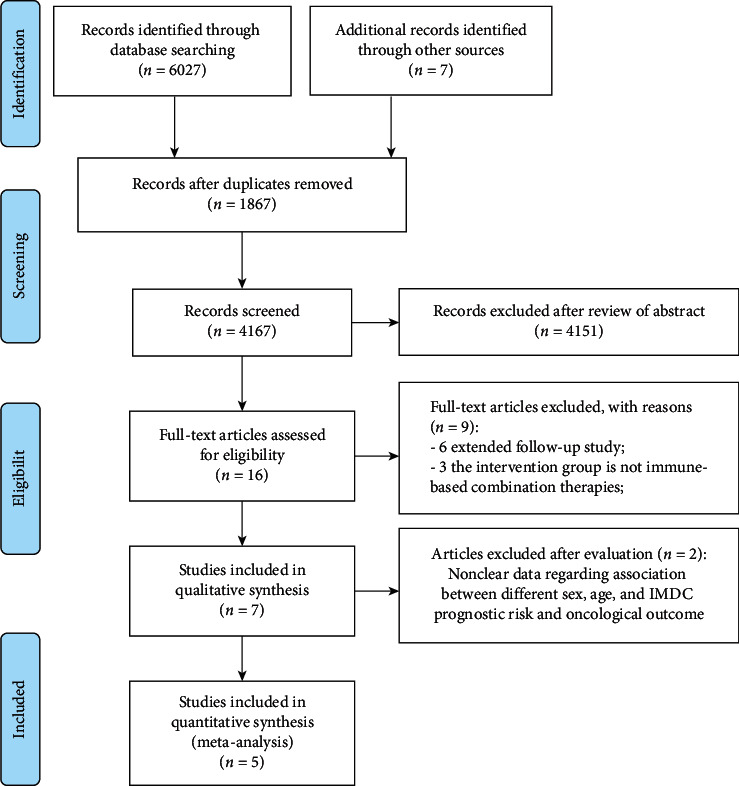
Flow diagram of the study selection procedure for the systematic review and meta-analysis.

**Figure 2:**
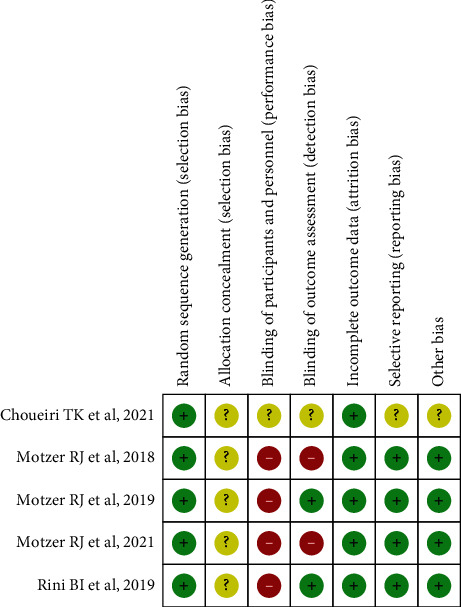
Summary of risk of bias in included studies.

The initial search strategy retrieved 6034 publications whose titles were screened for eligibility. After deleting duplicates, there were still 4167 studies, and 4151 reports were deleted during the abstract screening, where 16 articles were fully evaluated. Afterwards, a total of 5 RCTs involved in phase III (including 4206 participants) were found to be in accord with the inclusion standard and included in this analysis.  Figure 1 displays the flow chart for the search strategy. The baseline demographics were balanced in the immune-based combinations and control group among the included studies (Table 1). Through utilizing the seven-item criteria in RevMan 5.3, the assessment of the risk of bias between the two reviewers exhibited overall consistency (Figure 2).

### 3.2. Association of Sex with OS and PFS

**Figure 3:**
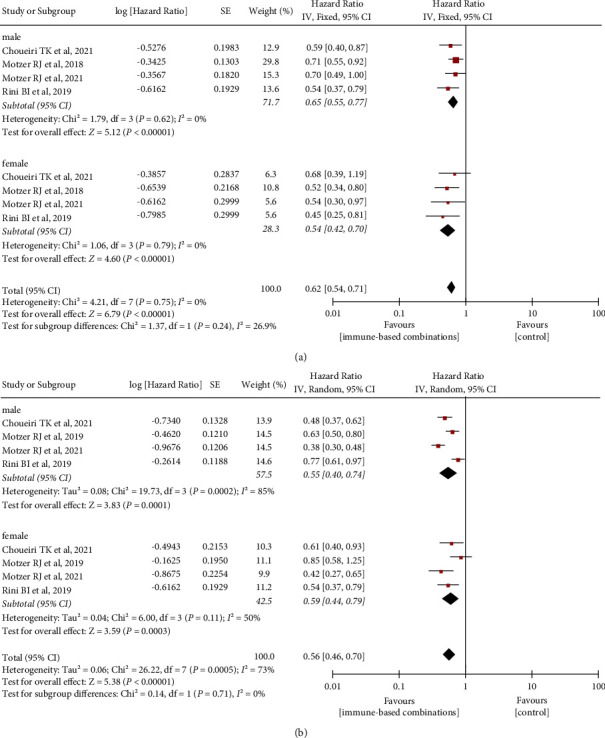
Differences in OS and PFS benefit associated with immune-based combinations in men and women by subgroups. (a) OS, overall survival; (b) PFS, progression-free survival. SE, standard error; IV, inverse variance; and CI, confidence interval.

All five studies were included in this meta-analysis and evaluated for the association between sex and OS/PFS among patients with mRCC. As shown in Figure 3, an OS and PFS superiority of immune-based combinations in comparison with control therapy was observed in both men (OS : HR 0.65, 95% CI 0.55–0.77; PFS : HR 0.55, 95% CI 0.40–0.74) and women (OS : HR 0.54, 95% CI 0.42–0.70; PFS : HR 0.59, 95% CI 0.44–0.79). There was no remarkable difference in OS and PFS from immune-based combinations over control therapy between men and women (OS: *P*=0.24, I^2^ = 26.90%; PFS: *P*=0.71, I^2^ = 0%). The I^2^ value in OS (men: *χ*^2^ = 1.79, *P*=0.62, I^2^ = 0%; women: *χ*^2^ = 1.06, *P*=0.79, I^2^ = 0%) revealed nonsignificant heterogeneity among these trials included. However, statistically significant heterogeneity was found in PFS for men (*χ*^2^ = 19.73, *P*=0.0002, I^2^ = 85%) and women (*χ*^2^ = 6.00, *P*=0.11, I^2^ = 50%), and heterogeneity was improved by excluding the study by Motzer et al. (men: *χ*^2^ = 7.04, *P*=0.03, I^2^ = 72%; women: *χ*^2^ = 2.90, *P*=0.23, I^2^ = 31%; Supplementary Figure 1) [12].

### 3.3. Association of Years with OS and PFS

**Figure 4:**
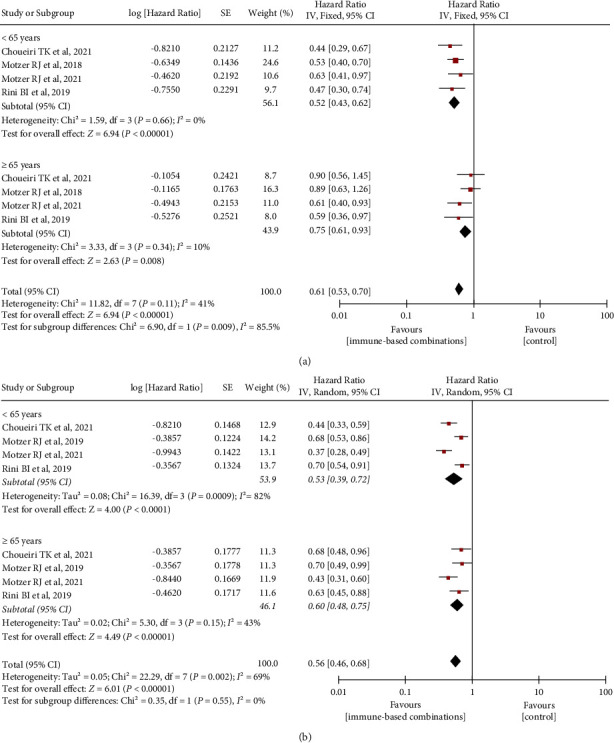
Differences in OS and PFS benefit associated with immune-based combinations in younger and older patients by subgroups. (a) OS, overall survival; (b) PFS, progression-free survival. SE, standard error; IV, inverse variance; and CI, confidence interval.

Five RCTs reported data on HR for OS and PFS according to patients' age. In contrast, to control treatment, the statistically significant superiority of immune-based combination therapies was found both in younger (<65 years: OS HR 0.52, 95% CI 0.43–0.62; PFS HR 0.53, 95% CI 0.39–0.72) and older (≥65 years: OS HR 0.75, 95% CI 0.61–0.93; PFS HR 0.60, 95% CI 0.48–0.75) patients (Figure 4). A remarkable difference in the OS from immune-based combinations in comparison with control therapy was found between the two age subgroups (*χ*^2^ = 6.90, *P*=0.009, I^2^ = 85.5%), while no significant difference was shown in PFS (*χ*^2^ = 0.35, *P*=0.55, I^2^ = 0%). The I^2^ value in OS (<65 years: *χ*^2^ = 1.59, *P*=0.66, I^2^ = 0%; ≥65 years: *χ*^2^ = 3.33, *P*=0.34, I^2^ = 10%) and PFS (≥65 years: *χ*^2^ = 5.30, *P*=0.15, I^2^ = 43%) displayed nonevident heterogeneity among these trials included. However, an obvious heterogeneity was found in PFS for younger patients (*χ*^2^ = 16.39, *P*=0.0009, I^2^ = 82%).

### 3.4. Association of IMDC Prognostic Risk with OS and PFS

**Figure 5:**
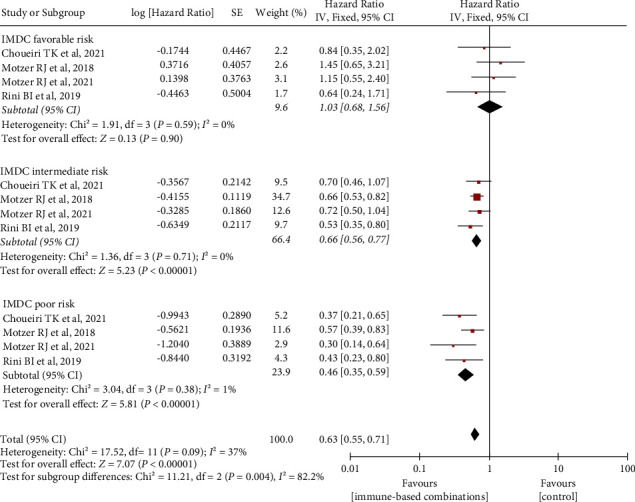
Differences in OS benefit associated with immune-based combinations in IMDC favorable-/intermediate-/poor-risk patients by subgroups. OS, overall survival. SE, standard error; IV, inverse variance; and CI, confidence interval.

**Figure 6:**
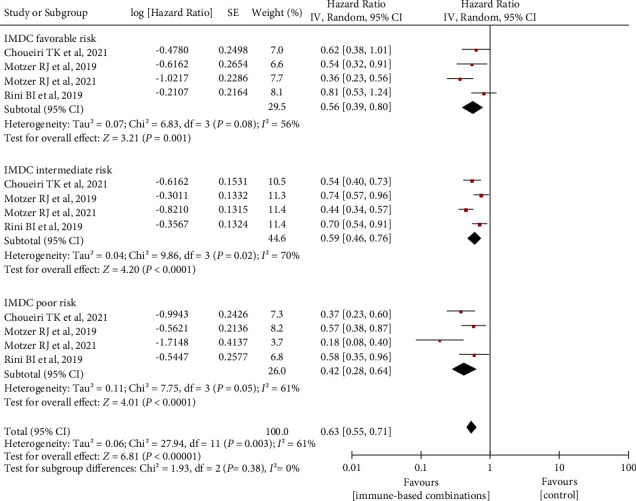
Differences in PFS benefit associated with immune-based combinations in IMDC favorable-/intermediate-/poor-risk patients by subgroups. PFS, progression-free survival. SE, standard error; IV, inverse variance; and CI, confidence interval.

A meta-analysis of five trials was performed to assess the outcomes of OS and PFS among mRCC patients with favorable/intermediate/poor-risk disease. As displayed in Figure 5, the findings suggested that in contrast to control treatment, immune-based combination therapy had evident OS superiority for both IMDC intermediate (HR 0.66, 95% CI 0.56–0.77) and poor-risk (HR 0.46, 95% CI 0.35–0.59) patients, while no significant difference was shown for patients with IMDC favorable-risk patients (HR 1.03, 95% CI 0.68–1.56). A remarkable difference in the OS from immune-based combinations in comparison with control therapy was found between the favorable/intermediate/poor-risk groups (*P* = 0.004, I^2^ = 82.2%). The I^2^ value (favorable: *χ*^2^ = 1.91, *P* = 0.59, I^2^ = 0%; intermediate: *χ*^2^ = 1.36, *P* = 0.71, I^2^ = 0%; poor: *χ*^2^ = 3.04, *P* = 0.38, I^2^ = 1%) displayed nonevident heterogeneity in trials included. Furthermore, as shown in Figure 6, PFS was improved markedly in mRCC patients with the immune-based combination therapies compared with control therapy (favorable: HR 0.56, 95% CI 0.39–0.80; intermediate: HR 0.59, 95% CI 0.46–0.76; poor: HR 0.42, 95% CI 0.28–0.64). The I^2^ value (favorable: *χ*^2^ = 6.83, *P* = 0.08, I^2^ = 56%; intermediate: *χ*^2^ = 9.86, *P* = 0.02, I^2^ = 70%; poor: *χ*^2^ = 7.75, *P* = 0.05, I^2^ = 61%) indicates a moderate heterogeneity, which was improved when the study by Motzer et al. was removed (favorable: *χ*^2^ = 1.52, *P* = 0.47, I^2^ = 0%; intermediate: *χ*^2^ = 2.64, *P* = 0.27, I^2^ = 24%; poor: *χ*^2^ = 2.25, *P* = 0.32, I^2^ = 11%) [12]. However, any differences in PFS were not also demonstrated between the favorable-/intermediate-/poor-risk groups (*P* = 0.38, I^2^ = 0%), despite exclusion of the trial by Motzer et al. (*P* = 0.17, I^2^ = 43.0%; Supplementary Figure 2).

### 3.5. Publication Bias

**Figure 7:**
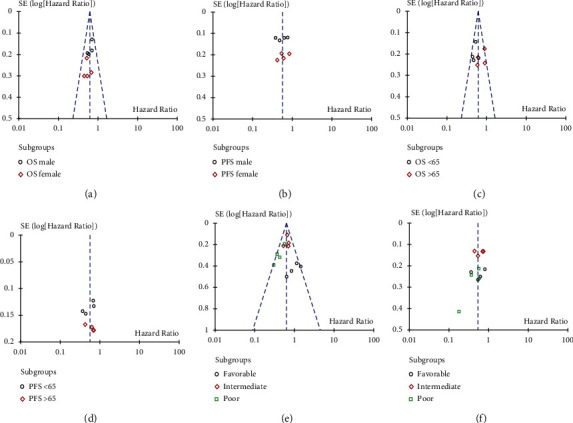
Funnel plot of the hazard ratio (HR) of overall survival (OS) and progression-free survival (PFS) in patients by different subgroups. (a) OS in patients with different sex; (b) PFS in patients with different sex; (c) OS in patients with different age; (d) PFS in patients with different age; (e) OS in patients with different IMDC prognostic risk; and (f) PFS in patients with different IMDC prognostic risk.

The publication bias of the primary outcomes (PFS and OS benefits from different sex, age, and IMDC prognostic risk patients) was assessed and represented using a funnel plot. As shown in Figure 7, the inverse funnel plot was approximately symmetric, thus the publication bias of this meta-analysis was well controlled and the reliability was satisfactory.

## 4. Discussion

In recent studies, immune-based combination therapies demonstrated a survival benefit for patients with mRCC, supporting the significance of these combinations as novel first-line treatment options for these patients. Compared with sunitinib, our meta-analysis suggested an overall trend for immune-based combination therapies providing preferable OS and PFS benefits in mRCC patients, regardless of age and gender. For the IMDC prognostic risk, our results showed that immune-based combinations improve OS in the intermediate-/poor-risk patients, except the favorable-risk patients, and a PFS benefit of immune-based combinations was found for all favorable/intermediate/poor-risk.

To our knowledge, this is the first research to clearly evaluate the efficacy of ICI combination with ICI/TT according to the mRCC patient's sex and years. In the aspect of the correlation between sex and the survival benefit of immune-based combinations, our findings were different from the meta-analysis by Conforti et al. [14], who reported an increased survival benefit of immunotherapy (a single ICI or combination with chemotherapy) in male versus female patients. The major reason that our study is derived from the comparison between the intervention group treated with ICI combination with ICI/TT and the control group that received targeted therapies may explain the conflicting results. Targeted therapies may present sex-based differential pharmacokinetics and therapeutic outcomes in RCC treatment [25, 26]. Furthermore, as has been observed in the research by Conforti et al. [14], women who participated in these clinical trials to evaluate immune-based combination therapies for mRCC patients were underrepresented, and so new clinical trials involving more women are needed in the future. For the correlation between years and the survival benefit of immune-based combination therapies, a meta-analysis by Nishijima et al. [27] indicated that ICIs markedly raised the OS of both younger and older patients in contrast to controls, and there was no evident difference between the two age groups. A benefit in PFS survival was not found in patients aged 65 years or older, whereas an improvement in PFS survival was observed in patients aged younger than 65 years. However, an OS and PFS benefit of immune-based combinations were found for both younger and older patients in our study, which showed a difference in the OS benefit associated with immune-based combinations in older vs. younger patients, while a homogeneous PFS benefit was detected between the two groups. This heterogeneity may be mainly associated with various types of cancer.

The IMDC risk scores use available clinical and laboratory information, including time from diagnosis to systemic therapy, Karnofsky performance status (KPS), hemoglobin concentration, calcium concentration, absolute neutrophil count, and platelet count, to prognosticate survival at the initiation of targeted therapies for mRCC patients. In the era of immune-based combination therapies as first-line therapy for mRCC, the significance of IMDC prognostic criteria remains to be determined. In recent National Comprehensive Cancer Network version 3.2022 guidelines [28], immune-based combination therapies including axitinib-pembrolizumab, cabozantinib-nivolumab, and lenvatinib-pembrolizumab were used as the preferred first-line therapy option for patients in both the favorable and poor/intermediate IMDC risk groups. For the ICI–ICI therapy, ipilimumab-nivolumab was only suitable for patients with poor/intermediate IMDC risk as the preferred first-line therapy option based on these data in the CheckMate 214 trial [6]. Data from the CheckMate 214 trial observed that the 18-month OS in poor-/intermediate-risk patients favored ipilimumab-nivolumab rather than sunitinib, whereas the exploratory analysis for the OS data displayed the opposite results for patients with the favorable-risk disease. In this meta-analysis, however, there was also no evidence of OS benefit among favorable-risk patients' response to the ICI-TT therapies in addition to ipilimumab-nivolumab.

Another interesting point is how the immune-based combinations differ in their characteristics of themselves and their survival benefits from mRCC subgroups. Pembrolizumab and nivolumab are the types of programmed death 1 (PD-1) inhibitors. Avelumab and ipilimumab inhibit the programmed death-ligand 1 (PD-L1) and cytotoxic T-lymphocyteantigen-4 (CTLA4), respectively. Lenvatinib (targeting VEGF receptors, FGFR1, PDGFR*α*, KIT, RET) showed a PFS benefit for patients who have progressed after receiving VEGF-targeted therapy such as sunitinib [29]. In accordance with the findings of this analysis, it appears that pembrolizumab-lenvatinib yielded the highest PFS in patients with different genders (male: HR 0.38, 95% CI 0.30–0.48; female: HR 0.42, 95% CI 0.27–0.65), years (<65 years: HR 0.37, 95% CI 0.28–0.49; ≥65 years: HR 0.43, 95% CI 0.31–0.60) and IMDC prognostic risk (favorable: HR 0.0.36, 95% CI 0.23–0.56; intermediate: HR 0.44, 95% CI 0.34–0.57; poor: HR 0.18, 95% CI 0.08–0.40). Cabozantinib (targeting AXL, MET, and VEGF receptors) resulted in significantly longer PFS compared with sunitinib [30]. Axitinib is a potent, selective, second-generation inhibitor of VEGFR 1, 2, and 3, which conferred PFS benefits in patients who had previously received sunitinib therapy [31]. Herein, favorable-risk populations did not significantly benefit from nivolumab-cabozantinib (HR 0.62, 95% CI 0.38–1.01) and pembrolizumab-axitinib (HR 0.81, 95% CI 0.53–1.24) compared to sunitinib. Furthermore, the extent of OS benefit differed among these immune-based combinations in a subgroup of patients. The results of this analysis showed that nivolumab-ipilimumab, the only ICI–ICI combination approved for the first-line treatment of mRCC, may not be best for OS for all subgroups, especially older (≥65 years) patients. In addition, nivolumab-cabozantinib did not provide significantly higher OS than sunitinib for females or older (≥65 years) or favorable-/intermediate-risk patients.

## 5. Strengths and Limitations

Compared with previous studies [32, 33] aiming to indirectly compare the efficacy and safety of first-line treatments or assess the predictive value of PD-L1 in mRCC patients treated with ICIs, we first evaluated the association of survival benefits from immune-based combination therapies in mRCC patients over first-line treatments with 3 variables: sex, age, and IMDC prognostic risk. Nevertheless, some limitations of this study should be considered. Firstly, several types of biases may limit the validity of the overall findings in this meta-analysis, such as publication bias, given that our analysis was performed according to the published literature. Moreover, some potential bias originated from inconsistencies in the patient characteristics, intervention regimens, and evaluation indexes. In addition, although all enrolled studies with a total of 4206 participants were phase III RCTs, the results were all open-label trials, and although there may be performance and detection bias, these data may not be entirely generalizable to real-world practice. Secondly, the OS and PFS benefits of immune-based combinations were not evaluated in all trials, which resulted in a lack of comprehensive evaluation among all existing treatments. Furthermore, the subgroup analyses related to comparisons between ICI–ICI, and ICI-TT were not investigated in our study owing to the small number of RCTs. Thirdly, the potential AEs of immune-based combinations related to excessive immune activation are essential for physicians managing patients with a variety of cancers. Appropriate management of toxicities associated with ICI requires early identification of underlying immune-related adverse events (irAEs) in order to administer adequate treatment [34]. A network meta-analysis by Quhal et al. [35] indirectly compared the safety profiles of various immune-based combinations that were evaluated in the first-line management of mRCC, who observed that the mortality related to the treatment of all the included combinations was commonly low, and there was no statistically significant difference in comparison with sunitinib, while the different rates of irAEs occurred in immune-based combinations. However, the irAEs involving different sex, age, and IMDC prognostic risk patients were not assessed, and the results may not be necessarily consistent. Finally, the results originated mostly from patients with clear cell histology, and thus, they may not be suitable for patients with other histologies.

## 6. Conclusions

An OS and PFS benefit of immune-based combination therapies were found for mRCC patients with different genders, ages (<65 vs. ≥65 years), and IMDC prognostic risks in this meta-analysis, except for IMDC favorable-risk patients in OS. The relative OS and PFS benefit from immune-based combinations over the control group is similar in patients of different sex. However, a significant difference in relative OS benefit from immune-based combinations was found in patients of different ages and IMDC prognostic risk. These findings suggest that immune-based combination therapies should not be restricted to certain mRCC patients in gender categories. However, patients' age and IMDC prognostic risk should be considered in the assessment of survival benefits as the significant variables predicting the relative benefit of immune-based combination therapies and guiding patients and clinicians to determine the personalized treatment strategies for patients with mRCC.

## Figures and Tables

**Table 1:** The characteristics of the included studies.

Included trials	Location (s); study design	Phase	Arms		Gender (male/female); mean age (range)		IMDC prognostic risk (favorable/intermediate/poor)	
			Treatment	Control	Treatment	Control	Treatment	Control
Motzer RJ et al, 2018;	Multinational; RCT	III	Nivolumab-ipilimumab	Sunitinib	413/137;	395/151;	125/334/91	124/333/89
NCT02231749					62 (26–85)	62 (21–85)		

Rini BI et al, 2019;	Multinational; RCT	III	Pembrolizumab- axitinib	Sunitinib	308/124;	320/109;	138/238/56	131/246/52
NCT02853331					62 (30–89)	61 (26–90)		

Motzer RJ et al, 2019;	Multinational; RCT	III	Avelumab- axitinib	Sunitinib	316/126;	344/100;	94/271/72	96/276/71
NCT02684006					62 (29–83)	61 (27–88)		

Choueiri TK et al, 2021;	Multinational; RCT	III	Nivolumab- cabozantinib	Sunitinib	249/74;	232/96;	74/188/61	72/188/68
NCT03141177					62 (29–90)	61 (28–86)		

Motzer RJ et al, 2021;	Multinational; RCT	III	Pembrolizumab- lenvatinib	Sunitinib	255/100;	275/82;	110/210/33	124/192/37
NCT02811861					64 (34–88)	62 (32–86)		

## Data Availability

All data relevant to the study are included in the article or uploaded as supplementary information.

## Supplementary Materials

Supplementary Materials Supplementary Table S1: the PRSIMA 2009 checklist. Supplementary Figure S1: heterogeneous analysis of the results about PFS benefit from immune-based combinations in men and women. Supplementary Figure S2: heterogeneous analysis of the results about PFS benefit from immune-based combinations in IMDC favorable/intermediate/poor-risk patients.
