# Supplementary material for: The Predictive Value of Three Variables in Patients with Metastatic Renal Cell Carcinoma Treated with Immune-Based Combination Therapies in Randomized Clinical Trials: A Systematic Review and Meta-Analysis
Source: J Oncol. 2022 Sep 10;2022:7733251. doi: 10.1155/2022/7733251 (PMC9482552; doi:10.1155/2022/7733251)
Supplement: Supplementary Materials — Supplementary Table S1: the PRSIMA 2009 checklist. Supplementary Figure S1: heterogeneous analysis of the results about PFS benefit from immune-based combinations in men and women. Supplementary Figure S2: heterogeneous analysis of the results about PFS benefit from immune-based combinations in IMDC favorable/intermediate/poor-risk patients. [file 7733251.f1.zip › 7733251.f1/Supplementary material (1).pdf]

| Study or Subgroup | log[Hazard Ratio] | SE | Weight | Hazard Ratio IV, Random, 95% CI |
|---|---|---|---|---|
| **1.2.1 male** | | | | |
| Choueiri TK et al, 2021 | -0.7340 | 0.1328 | 19.6% | 0.48 [0.37, 0.62] |
| Motzer RJ et al, 2019 | -0.4620 | 0.1210 | 21.1% | 0.63 [0.50, 0.80] |
| Rini BI et al, 2019 | -0.2614 | 0.1188 | 21.4% | 0.77 [0.61, 0.97] |
| Subtotal (95% CI) | | | 62.1% | 0.62 [0.48, 0.80] |
| Heterogeneity: Tau² = 0.04; Chi² = 7.04, df = 2 (P = 0.03); I² = 72% | | | | |
| Test for overall effect: Z = 3.58 (P = 0.0003) | | | | |
| **1.2.2 female** | | | | |
| Choueiri TK et al, 2021 | -0.4943 | 0.2153 | 11.5% | 0.61 [0.40, 0.93] |
| Motzer RJ et al, 2019 | -0.1625 | 0.1950 | 13.1% | 0.85 [0.58, 1.25] |
| Rini BI et al, 2019 | -0.6162 | 0.1929 | 13.3% | 0.54 [0.37, 0.79] |
| Subtotal (95% CI) | | | 37.9% | 0.66 [0.50, 0.86] |
| Heterogeneity: Tau² = 0.02; Chi² = 2.90, df = 2 (P = 0.23); I² = 31% | | | | |
| Test for overall effect: Z = 3.03 (P = 0.002) | | | | |
| **Total (95% CI)** | | | **100.0%** | **0.63 [0.53, 0.75]** |
| **Heterogeneity: Tau² = 0.02; Chi² = 10.05, df = 5 (P = 0.07); I² = 50%** | | | | |
| **Test for overall effect: Z = 5.10 (P < 0.00001)** | | | | |
| **Test for subgroup differences: Chi² = 0.09, df = 1 (P = 0.76), I² = 0%** | | | | |

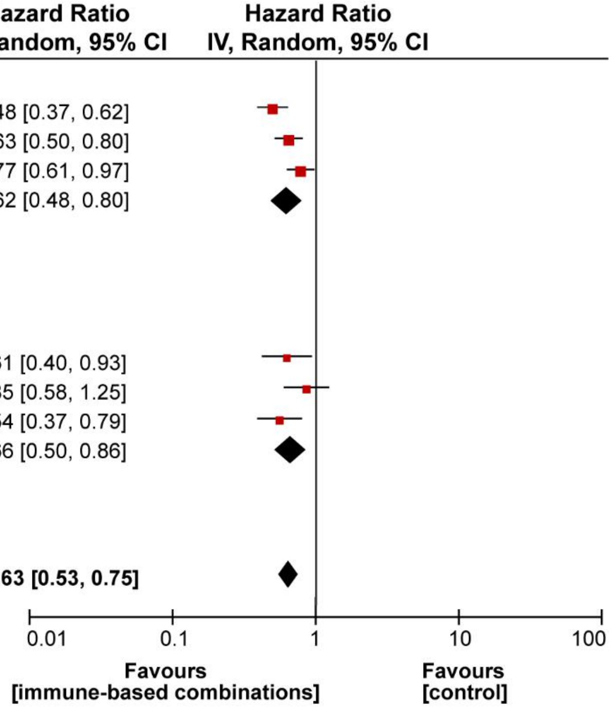


**Supplementary Figure 1. Heterogeneous analysis of the results about PFS benefit from immune-based combinations in men and women.** SE, standard error; IV, inverse variance; CI, confidence interval.

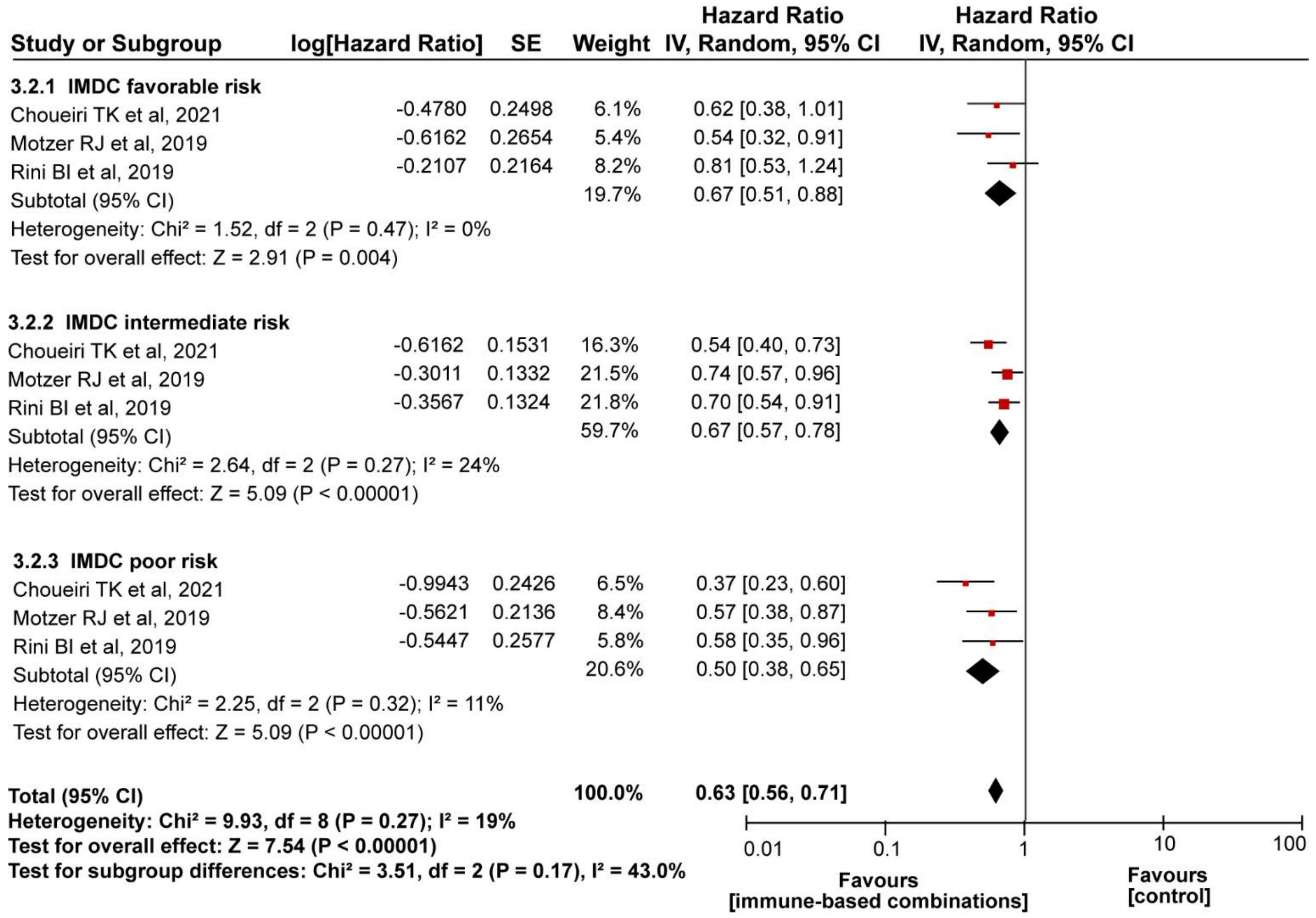


**Supplementary Figure 2. Heterogeneous analysis of the results about PFS benefit from immune-based combinations in IMDC favorable/intermediate/poor-risk patients.** SE, standard error; IV, inverse variance; CI, confidence interval.
